# In Vitro and In Vivo Comparative Evaluation of a Shellac-Ammonium Paclitaxel-Coated Balloon versus a Benchmark Device

**DOI:** 10.1155/2021/9962313

**Published:** 2021-05-25

**Authors:** Congying Xia, Yunhan Jiang, Shuangshuang Li, Dan Xiong, Xiaojie Chen, Yufang Chen

**Affiliations:** ^1^Department of Cardiology, West China Hospital of Sichuan University, Chengdu, China; ^2^Department of Cardiovascular Surgery, West China Hospital of Sichuan University, Chengdu, China; ^3^Laboratory of Human Diseases and Immunotherapies, West China Hospital of Sichuan University, Chengdu, China; ^4^Institution of Immunology and Inflammation, Frontiers Science Center for Disease-Related Molecular Network, West China Hospital of Sichuan University, Chengdu, China; ^5^Department of R & D, Cardionovum Co, Ltd, Wuhan, China; ^6^Department of Medical Affair, Cardionovum Co, Ltd, Wuhan, China

## Abstract

**Objectives:**

The present study was designed to compare the characteristics and performance regarding drug delivery of a novel drug-coated balloon (DCB) to a benchmark device (Restore® versus SeQuent® Please) in an in vitro and in vivo model.

**Background:**

Although Restore® and SeQuent® are both paclitaxel-coated, they use different coating excipient, shellac-ammonium salt and iopromide, respectively. Preclinical study comparing these two different commercial DCBs regarding their characteristics and effects on early vascular response is sparse.

**Methods:**

Restore® and SeQuent® DCBs were scanned with electron microscopy for surface characteristic assessment. Both DCBs were transported in an in vitro vessel model for the evaluation of drug wash-off rate and particulate formation. Eighteen coronary angioplasties with either Restore® or SeQuent® DCBs were conducted in 6 swine (three coronary vessels each). Histopathological images of each vessel were evaluated for vessel injury.

**Results:**

The surface of Restore® DCB was smooth and evenly distributed with hardly visible crystal, while SeQuent® DCB showed a rougher surface with relatively larger apparent crystals. Restore® DCB had a lower drug wash-off rate and fewer large visible particles, compared to the SeQuent® DCB. No significant difference in mean injure score was found between Restore® and SeQuent® group.

**Conclusion:**

Our results suggest that Restore® is better in preclinical performance regarding less release of particles and lower drug wash-off rate as compared to SeQuent® Please. The Restore® DCB, using stable amorphous coating and shellac-ammonium salt as an excipient, appears to provide an advantage in drug delivery efficacy; however, further clinical studies are warranted.

## 1. Introduction

Drug-coated balloons (DCBs) have been developed for the percutaneous coronary angioplasty and recommended in treatment of in-stent restenosis [1]. Prior clinical studies have proved the clinical efficacy and safety of several commercial DCBs in treatment of bare metal stent restenosis [2–4]. In common, these DCBs are all coated with the antiproliferative drug, paclitaxel, which can inhibit neointimal hyperplasia and consequently prevent or delay in-stent restenosis [5]. However, the considerable difference in drug carrier among the currently commercial DCBs is of great importance, because it can affect the safety and efficacy. SeQuent® Please, a typical benchmark DCB, uses the iopromide drug coating technology to provide a targeted paclitaxel release in vessel lumen. Its safety and efficacy have been investigated by prior in-human studies [6, 7]. Restore® is a novel DCB device that has been recently introduced in the market, with the same drug load of paclitaxel, but it uses a different coating excipient, namely, the shellac-ammonium salt, a balanced-hydrophilic coating technology. Few preclinical data have been published regarding the safety and efficacy of this new device. Especially, to the best of our knowledge, there is no preclinical study comparing these two different commercial DCBs regarding the effects of different coating systems on early vascular response.

Therefore, the current study aimed to compare the characteristics and performance regarding drug delivery of a novel DCB to a benchmark device (Restore® versus SeQuent® Please) in an in vitro and in vivo model.

## 2. Materials and Methods

### 2.1. In Vitro Experiments

#### 2.1.1. Scanning Electron Microscopy Assessment

Morphologies of two compared DCBs were evaluated in a scanning electron microscopy system (Quanta FEG 450, FEI Inc., Hillsboro) as described previously [8]. In brief, we obtained the balloon segments of Restore® (Cardionovum GmbH, Germany) and SeQuent® Please (B. Braun Melsungen AG, Germany) DCBs by carefully cutting them from the catheter with scissors. We then attached the balloon samples to the specimen mounted and examined the balloon samples for different coating positions.

#### 2.1.2. Drug Loss and Paclitaxel Quantification

We used an in vitro vessel model to mimic the transfer process of two compared DCBs (*n* = 3 per group) in the vessel to assess the drug loss rate during tracking. The detailed methods of model preparation have been described previously [9]. Each tested DCB was manually advanced over the guide wire through the guide catheter into the silicone tube that was fully surrounded by 0.9% sodium chloride. The balloon was left noninflated in the silicone tube for 2 minutes and then withdrawn. We then cut the balloon for the determination of residual drug load. The cut sample was jolted for 30 seconds in the ultrasound to ensure that residual paclitaxel attached to the balloon was completely dissolved in ethanol. The amount of paclitaxel in the collected solution was quantified using the high performance liquid chromatography tandem mass spectrometry (HPLC-MS). A total of 10 *μ*L of the test solutions was injected into a column (Ultimate LP-C18, 5 *μ*m, 4.6 × 250 mm, Welch Materials Inc., Shanghai, China). Detailed chromatographic conditions were as follows: UV detection at 227 nm, flow rate of 1.0 mL/min, and column temperature of 30°C, with calibrated measurement range of 4–200 ug/mL. The drug wash-off rate was calculated based on the following formula: (total drug load − residual drug load)/total drug load × 100%.

#### 2.1.3. Particulate Formation Study

An in vitro coating particulate formation test was conducted to assess the coating characterization. Each DCB was transported into an individual tube circulated with the media of 0.9% sodium chloride through a 6F guide catheter. We then inflated the compared DCBs per their nominal label for 60 seconds. After deflation, the DCBs were withdrawn, and the media was collected, filtered (with the 0.22 *μ*m pore size filter), dried, and then imaged (4×) using the Olympus microscope (IX73P1F, Olympus Life Science Inc., Tokyo, Japan). Qualitative analysis of the amount particles was performed with the Clemex Vision Particle Analyzer (Longueuil, Quebec, Canada). We highlighted all particulates with green color for better visualization and performed a qualitative assessment of particular images for the remaining insoluble particulate.

### 2.2. In Vivo Experiments

This study protocol was approved by the Institutional Animal Care and Use Committee of Animal Laboratory Center of Nongnong Biotechnology Co., Ltd., and all animals received standard care complying with the guidelines of the Institute of Laboratory Animal Resources [10].

#### 2.2.1. Grouping and Devices

A total of six healthy young adult swine (30 weeks, weighted 30–40 kilograms) were included in this study and were acclimated for at least 3 days. Animals were randomly allocated into two treatment groups, Restore® group (*n* = 3) versus SeQuent® Please group (*n* = 3). The Restore® DEB contained paclitaxel at a concentration of 3.0 *μ*g/mm^2^ balloon surface using shellac-ammonium salt as excipient. The SeQuent® Please also contained paclitaxel at a concentration of 3.0 *μ*g/mm^2^ balloon surface but using iopromide as excipient. The sizes of these two compared DEB systems were both 2.75 mm (diameter) × 20 mm (length).

#### 2.2.2. Interventional Procedures

Animals were given 100 mg aspirin and 75 mg clopidogrel daily 3 days prior to the coronary balloon angioplasty and for the remainder of the study until being sacrificed (28 days following coronary balloon angioplasty). Animals were put under general anesthesia, intubated, and supported with mechanical ventilation. Angiographic images were acquired to identify the appropriate location for balloon injury. DCBs were then placed to the identified target area (1.1 : 1.3 balloon-to-artery ratio), inflated, and held for a total of 60 seconds. For each animal, coronary balloon angioplasty was conducted in all three coronary arteries, namely, left descending artery, circumflex artery, and right coronary artery, resulting in a total of 18 injury lesions. Twenty-eight days later, terminal angiography was performed for the assessment of all treated coronary vessels.

#### 2.2.3. Tissue Harvest and Histopathological Assessment

Animals were put under euthanasia 28 days after coronary balloon angioplasty, and a complete necropsy was performed. Coronary arterial samples were harvested and processed according to the standardized methodologies described previously [11]. Segments with location of 5 mm proximal to the site of coronary balloon angioplasty were treated as the final specimens. Specimens were then stained with hematoxylin and eosin (H&E) after fixation for the determination of coronary artery injury. We then quantitatively assessed the vessel injury by calculating an injury score using Schwartz's method [12]. According to the different extent of injury description, a score of 0 to 3 was given to each sample, namely, score 0: internal elastic lamina intact, endothelium typically denuded, and media compressed but not lacerated; score 1: internal elastic lamina lacerated and medial typically compressed but not lacerated; score 2: internal elastic lamina lacerated, medial visibly lacerated, and external elastic lamina intact but compressed; and score 3: external elastic lamina lacerated, typically large lacerations of media extending through the external elastic lamina, and coil wires sometimes residing in adventitia. A mean injury score was then calculated for each DCB device group.

#### 2.2.4. Drug Transfer to the Coronary Vessel Wall

Six swine received coronary balloon angiography (*n* = 3 per group) to determine the uptake of paclitaxel into the vessel wall. An uncoated balloon was first dilated in the target vessel, followed by a postdilatation with DCB at the same site, where the DCB was inflated and held for a total of 60 seconds. In each animal, balloon angioplasty was performed at left anterior descending coronary artery and right coronary artery. After 60 minutes, treated coronary segments were harvested to test the uptake of drug content. Paclitaxel was then extracted from the harvested samples with dimethyl sulfoxide. Collected samples were analyzed using HPLC-MS. Detailed chromatographic conditions have been described above in the section of In Vitro Experiments. Results were expressed by dividing the amount of paclitaxel that was transferred to the coronary vessel wall with the mass of collected tissue samples (*μ*g/g), in order to minimize the heterogeneity during sample collection.

### 2.3. Statistical Analysis

All statistical analyses were conducted using the statistical software Prisma Graphics (version 7, GraphPad Software, California). Continuous variables were presented as mean and standard variation. Continuous variable was first tested for normality using the Kolmogorov-Smirnov test. Mann-Whitney *U* test was used to compare the differences in drug wash-off rate and injury score between two independent groups. A two-tailed *p* value of 0.05 was considered as statistically significant.

## 3. Results

### 3.1. Comparison of Surface Morphology

**Figure 1:**
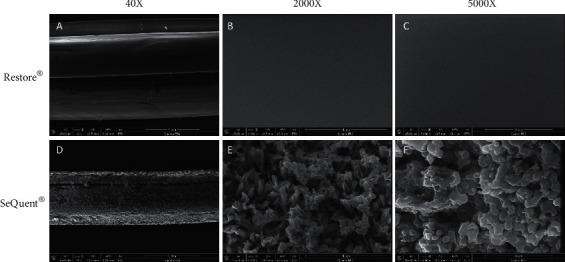
Representative electron microscopical images of Restore® versus SeQuent® Please balloon. Coating integrity of Restore® ((a) 40X, (b) 2000X, and (c) 5000X) balloons was complete, and the surface was smooth and evenly distributed with hardly visible crystal. SeQuent® Please ((d) 40X, (e) 2000X, and (f) 5000X) balloons had a rougher surface with relatively larger apparent crystals. Scale bars are 1 mm (a, d), 3 *μ*m (b, e), and 10 *μ*m (c, f).

We assessed the morphological surface of different commercial DCBs by using the electron microscopy. Representative figures showed heterogeneous coating for these two compared DCBs (Figure 1). More specifically, the coating integrity of the Restore® balloon was complete, and the surface was smooth and evenly distributed with hardly visible crystal. In contrast, the SeQuent® Please balloon showed a rougher surface with relatively larger apparent crystals.

### 3.2. In Vitro Drug Loss during Simulated Use

**Figure 2:**
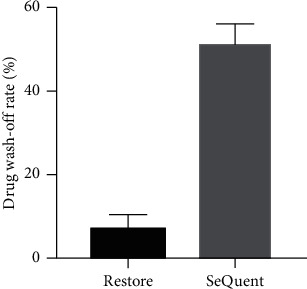
Results of quantitative analysis of drug wash-off rate. Drug wash-off rate of the Restore® group was substantially lower than that of the SeQuent® Please group (7% ± 6% versus 51% ± 9%, *p* < 0.001).

We further investigated the drug loss rate of different commercial DCBs during simulated process of DCB angioplasties. We found that the drug wash-off rate of the Restore® group was substantially lower, compared to the SeQuent® Please group (7% ± 6% versus 51% ± 9%, *p* < 0.001, Figure 2).

### 3.3. Particulate Formation Study

**Figure 3:**
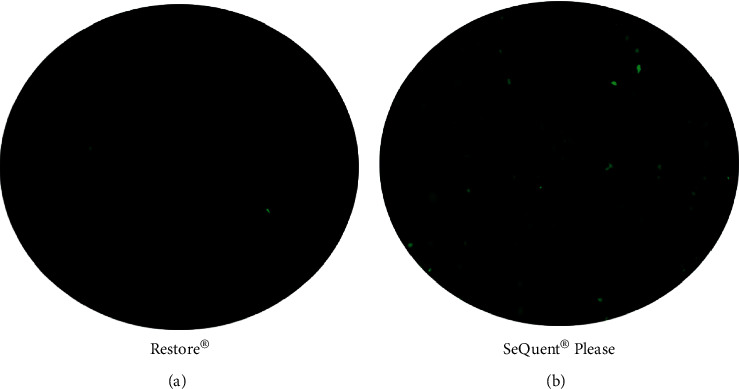
Representative images of particulate formation after balloon inflation. Restore® balloon (a) had fewer large visible particles compared to the SeQuent® Please balloon (b).

We qualitatively assessed the particulate formation of two compared DCBs by using the optical microscopy. Presentative figures showed that the Restore® DCB had fewer large visible particles than the SeQuent® Please DCB (Figure 3).

### 3.4. Histopathological Assessment

**Figure 4:**
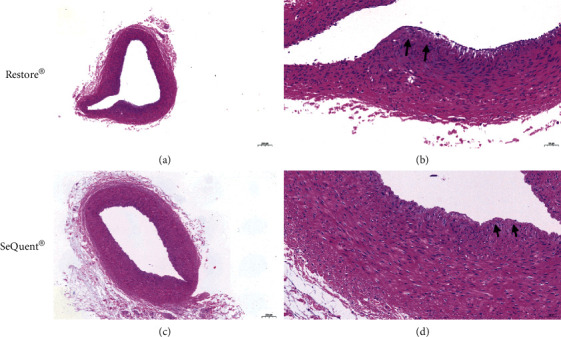
Representative histopathological images of coronary arteries. Cross-sectional vessel segment of an animal from Restore® group (a, b) and SeQuent® Please group (c, d) showing mild internal elastic lamina lacerated (black arrow). Scale bars are 200 *μ*m (a, c) and 50 *μ*m (b, d).

A total of 18 DCB angioplasties were performed successfully in three major coronary arteries of 6 swine (Restore® group, *n* = 3, versus SeQuent® Please group, *n* = 3). There was no significant difference in mean injury score between Restore® group and SeQuent® Please group (0.78 ± 0.51 versus 0.78 ± 0.51, *p*=0.999). Coronary artery injury with proliferation of smooth muscle cells and accumulation of inflammatory cells at the site of treatment were observed after DCB angioplasties. However, these histopathological changes did not differ substantially between two treatment groups (Figure 4).

### 3.5. In Vivo Drug Transfer to the Vessel Wall

**Figure 5:**
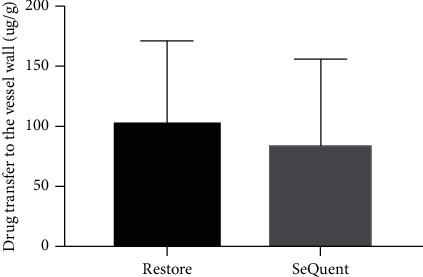
Paclitaxel transfer to the vessel wall in vivo experiments mimic coronary balloon angioplasty. Drug transfer to the vessel wall was comparable between Restore® and SeQuent® Please group (101.3 ± 63.5 *μ*g/g versus 83.9 ± 65.5 *μ*g/g, *p*=0.948).

We assessed the amount of paclitaxel which was transferred and penetrated to the coronary vessel wall (3 animals, 6 vessel samples per group). In the Restore® group, the uptake of paclitaxel by coronary vessel wall was slightly higher than that in the SeQuent® Please group, but it did not reach a statistically significant level (101.3 ± 63.5 *μ*g/g versus 83.9 ± 65.5 *μ*g/g, *p*=0.948, Figure 5).

## 4. Discussion

The current study aimed to compare the characteristics and preclinical performance of a novel DCB to a benchmark device (Restore® versus SeQuent® Please) in an in vitro and in vivo porcine model. As a consequence of the different coating technique, substantial differences in morphological surfaces were observed between the Restore® and SeQuent® Please DCBs. The Restore® DCB had fewer large visible particles and lower drug wash-off rate than the SeQuent® Please DCB. In spite of different coating properties, the coronary artery intimal healing without hyperplasia based on histopathological assessment was similar in these two DCBs.

In the field of percutaneous coronary intervention, DCB angioplasty has emerged as an alternative therapeutic strategy especially in myocardial infarction patients [13, 14] and a routine treatment for in-stent restenosis [15, 16]. Although paclitaxel has been considered as the coated antiproliferative drug of choice for DCBs, the development of DCB is a complex procedure involving different techniques, for example, how the drug is attached to the balloon surface and what the type of crystal formation is. Both these above-mentioned aspects can determine the efficacy of DCBs, because they play roles in the time the drug can be held during delivery and the time it takes to transfer the drug into the vessel walls and consequently impact the residual amount of drug that can be delivered to the target lesion. The SeQuent® Please balloon is a benchmark device that has been widely used in clinical practice. It is coated with a patented drug matrix, on which hydrophilic iopromide is embedded with paclitaxel. This technique allows the transfer of paclitaxel onto the tissues of coronary artery wall through a hydrophilic environment [17]. However, a disadvantage of this coating matric is that its hydrophilic characteristic cannot guarantee an entire drug transfer during delivery. Nonnegligible drug wash-off rates (up to 42%) of the SeQuent® Please DCB have been reported in prior studies [18, 19]. To overcome this shortcoming, a novel device, the Restore® DCB with a SAFEPAX stable amorphous coating and using shellac-ammonium salt as the excipient, has been introduced. In this study, we found that the drug wash-off rate of the Restore® group was substantially lower, compared to the SeQuent® Please group. This result supports that the use of shellac as a carrier can be more suitable.

Besides the DCB performance regarding drug loss, we first investigated the coating characteristic, namely, coating morphology. We found that the coating surface of Restore® DCB was smoother than that of the SeQuent® Please DCB. This can be a potential explanation of the less drug loss during delivery, since a rougher surface may lead to an increased friction during transit.

Furthermore, the safety aspect of DCB was assessed by the particulate formation study and histopathological assessment. Generation of particulars during DCB delivery may be associated with postprocedural complications such as downstream microembolization [20]. In the current study, we observed that the Restore® DCB had fewer large visible particles than the SeQuent® Please DCB. In addition, we compared the different responses to injury between these two compared DCBs. Our study showed comparable results of coronary artery injury as assessed by histopathological based scores. These results may suggest that Restore® DCB has a lower risk of potential downstream microembolization and better safety.

The limitations of this study need to be addressed. First, we only used healthy porcine coronaries free of atherosclerotic lesions or restenosis for the in vivo experiments. Therefore, it is not feasible for us to draw a conclusion on the efficacy and safety of the Restore® DCB as a treatment for the mimicked setting of in-stent restenosis. Second, we only compared the Restore® DCB with a single commercial device that has been widely used in the clinical practice. Whether the performance of the Restore® DCB is similar to or better than those of other devices remains to be further investigated.

## 5. Conclusion

In conclusion, Restore® has been shown to be better in preclinical performance regarding less release of particles and lower drug wash-off rate as compared to a benchmark device. Although further clinical studies are needed to compare the clinical efficacy and prognostics of different coating techniques, the Restore® DCB, using stable amorphous coating and shellac-ammonium salt as excipient, appears to provide an advantage in drug delivery efficacy.

## Data Availability

The data used to support the findings of this study are available from the corresponding author, Yufang Chen, upon reasonable request.
